# Quaternized cellulose and graphene oxide crosslinked polyphenylene oxide based anion exchange membrane

**DOI:** 10.1038/s41598-019-45947-w

**Published:** 2019-07-02

**Authors:** Gautam Das, Bang Ju Park, Jihyeon Kim, Dongho Kang, Hyon Hee Yoon

**Affiliations:** 1 Department of Chemical and Biological Engineering, Gachon University, Seongnam, Gyeonggi-do 13120 Republic of Korea; 2 Department of Electronic Engineering, Gachon University, Seongnam, Gyeonggi-do 13120 Republic of Korea

**Keywords:** Polymers, Nanocomposites

## Abstract

Anion exchange membrane fuel cells (AEMFCs) have captivated vast interest due to non-platinum group metal catalysts and fuel flexibility. One of the major shortcomings of AEMFCs, however, is the lack of a stable and high anion conducting membrane. This study introduces a new strategy for fabrication of high conducting anion exchange membrane (AEM) using a hybrid nanocomposite of graphene oxide (GO), cellulose, and poly(phenylene oxide) (PPO), which are functionalized with 1,4-diazabicyclo[2.2.2]octane. The compositional ratio of GO/cellulose/PPO was optimized with respect to ionic conductivity, water uptake, swelling ratio, and mechanical properties. The membrane at GO/cellulose/PPO weight ratio of 1/1/100 displayed an impressive hydroxyl conductivity of ∼114 mS/cm at 25 °C and ∼215 mS/cm at 80 °C, which is considerably higher than the highest value reported. Further, the hybrid composite membranes were mechanically stable even when operating at high temperature (80 °C). The result indicates that the introduction of quaternized GO and cellulose into a polymer matrix is a promising approach for designing high performance AEMs.

## Introduction

Over the past few years fuel cell technologies utilizing a polymer membrane electrolyte have been extensively investigate due to their zero greenhouse gas emissions, low operating temperature, and fuel flexibility^1–3^. The most well-known low temperature-fuel cell is the proton exchange membranes fuel cell (PEMFC)^4,5^, which has substantially a higher power output owing to the highly proton conducting membrane such as Nafion. Recently, PEMFC has been used in automobile sector; however, its wide scale commercialization still faces scrutiny owing to expensive catalyst systems, hydrogen infrastructure and high fuel crossover^2,6–8^. The use of Pt-based catalytic system constitutes nearly 50% of the cost of the PEMFC fabrication. In contrast, anion exchange membrane fuel cells (AEMFCs) operating at alkaline pH utilizes non- platinum based catalysts, providing better economic choice of catalytic materials^4,9^. Although AEMs currently offer several advantages over their PEM counterparts, including cheaper fabrication and fuel flexibility, most AEMs still suffer from poor chemical stability and low ion conductivity. Hence, much effort on the development of AEMs has been performed for the implementation of AEMFCs. An ideal AEM should possess high hydroxide conductivity, adequate chemical/mechanical stability in high pH solutions at elevated temperatures^10^. A high ionic conduction in AEMs was reported to be achieved by increasing the charge carriers in polymer matrix^4,5^. However, an increase in the charge capacity is accompanied with excessive water uptake which will ensue in membrane fragility at a high operating temperature (>60 °C). Thus, currently, studies on new polymer structures^11^, various cationic functional groups as charge carriers^4,12–14^, and blends or composite membranes^15–18^ have been performed to improve the AEM’s stability and anion conductivity. Various fillers for the composite AEMs such as ZrO_2_^19^, SiO_2_^20^, CNTs^21^, graphene oxide (GO)^22^ have been reported with concomitant enhancement in performance.

In this study, we proposed a novel hybrid composite membrane having two different fillers viz., cellulose and graphene oxide. Cellulose, which is a benign polymer, is an alternative to synthetic polymers due to its low density, high strength, large aspect ratio, and biocompatibility^23^. The abundant hydroxyl groups in cellulose provide opportunity to fine tune with functionalities to suit numerous applications. In case of polymer composites, such functionalization would enhance the compatibility of cellulose with the polymer matrix. Thus, judicially incorporating functionalized cellulose can impart ion conducting path as well as high bound water substantially enhancing the ionic conductivities of AEM^24^. Another nanomaterial that has captivated researchers is GO. There are few reports on utilization of GO as AEM materials with concomitant enhancement in the membrane performance^7,25^. The chemical functionalization of GO has been found to be a feasible and effective means of improving the dispersion of graphene similar to that observed for functionalized carbon nanotube-based composites^26^. Several researchers have reported that by functionalization of the fillers it is possible to enhance the ionic conductivity by just adding a small amount (0.05–1 wt%)^25,27–29^. Functionalized fillers such as cellulose and GO will inevitably increase the water uptake of the membranes owing to their hydrophilic nature. However, without sufficient interaction with the polymer matrix, these fillers might aggregate and cause deterioration in the membrane performance.

Recently, few studies have employed crosslinking to enhance the mechanical and ionic conductivities of AEMs^28,30,31^. Hossain *et al*.^32^ crosslinked brominated-poly-(2,6-dimethyl-phenylene oxide with dimethylamine. The AEMs demonstrated outstanding durability and alkaline stability at high temperature. A self-crosslinked poly(ethersulfone) containing benzyl bromide groups was also reported to enhance the ionic conductivity and stability. Nonetheless, there exist a tradeoff between stability and hydroxide conductivity, and research on this matter is still in the nascent stages.

We functionalized GO and cellulose by quarternization with 1,4-diazabicyclo[2.2.2]octane and subsequently crosslinked with quarternized PPO (QPPO) to obtain a multi-component composite membrane. Since cellulose and GO possess heterogeneous structures with that of the base PPO polymer, the induction of this hybrid nanofiller will endow phase segregation at the nanoscale with establishing contact points which can be connected through cross-linking with the polymer chains. Thus, it is believed that the covalent bonding will enhance the compatibility between GO, cellulose, and PPO resulting in stable and oriented microstructures while maintaining good thermal, mechanical and high anion conductivity properties. The hybrid composite membranes were synthesized and compositional ratio of GO/cellulose/PPO was optimized with respect to ionic conductivity, water uptake, swelling ratio, and mechanical properties.

## Result and Discussions

### Characterizations

**Figure 1:**
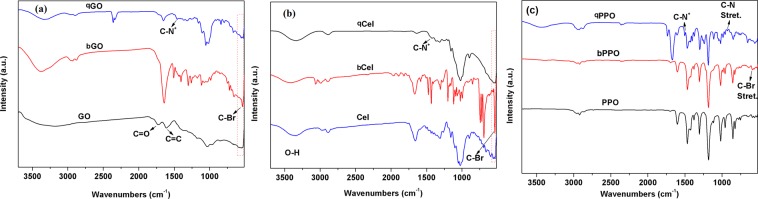
FTIR spectra of functionalized graphene oxide (**a**), cellulose (**b**), and PPO. (Cel = cellulose, bCel = brominated cellulose, qCel = quaternized cellulose, GO = graphene oxide, bGO = brominated graphene oxide, qGO = quaternized graphene oxide).

The FTIR spectra of brominated and quaternized cellulose and GO are shown in Fig. 1. The covalent bonding of bromine with cellulose and GO was proved by the appearance of C-Br symmetric stretching band at 541 cm^−1^ for bCel and at 539 cm^−1^ for bGO^33^. The C-Br stretching bands for qCel and qGO diminished upon quaternization. In addition, the appearance of C-N^+^ stretching band at around 1470 cm^−1^ (Fig. 1a) and 1463 cm^−1^ (Fig. 1b) for qCel and qGO, respectively, indicated the successful quaternization reaction^34^. Further, the –OH stretching band in both GO (3303 cm^−1^) and Cel (3354 cm^−1^), were shifted to higher wavenumber, indicating coordination with bromide and nitrogen moiety of both brominated and quaternized GO and Cel, respectively. Similarly, Fig. 1c shows the FTIR spectra of PPO, bPPO, and qPPO. The C-Br stretching vibration band was observed at 591 cm^−1^ for bPPO. The C-Br band diminished for qPPO, suggesting the successful attachment of DABCO following Menshutkin reaction^35^.

**Figure 2:**
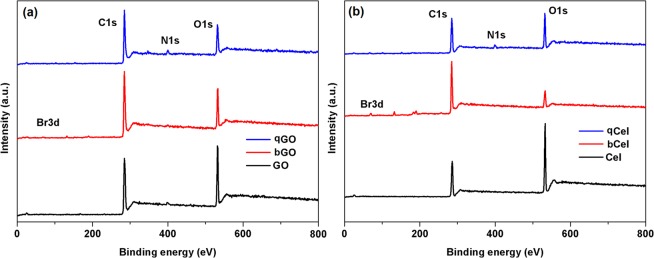
XPS survey spectrum (**a**) GO, and (**b**) Cel.

**Figure 3:**
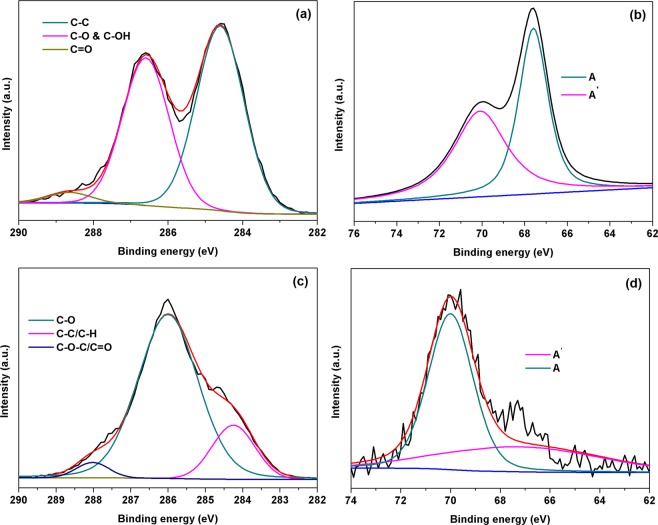
(**a**) C1s peak of GO, (**b**) high resolution 3d peak of bGO, (**c**) C1s peak of Cel and, (**d**) high resolution 3d peak of bCel, respectively.

**Figure 4:**
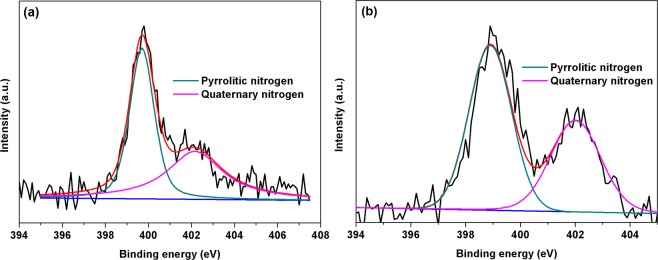
A **c**onvoluted spectrum of (**a**) N1s peak of qGO and (**b**) N1s peak of qCel.

The binding energy (B.E.) plots of the functionalized GO and cellulose is shown in Figs 2–4. The survey spectrum of functionalized GO, and cellulose is shown in Fig. 2. The B.E. plots shows two prominent peaks corresponding to C1s and O1s for GO (Fig. 2a) and cellulose (Fig. 2b). Additionally, the B.E. plots of bGO and bCel exhibited new peaks for Br 3d (Fig. 2) while qGO and qCel showed peaks corresponding to N1s. The deconvulated C1s of GO as shown in Fig. 3(a) shows three prominent peaks assigned to sp^2^ carbon (284.4 eV), C-O for epoxy/hydroxyl (286.2 eV) and C=O for carbonyl (287.2 eV)^36^. In the case of bGO (Fig. 3b) a high-resolution scan of Br 3d revealed a peak maximum at 70.07 eV assigned for covalently bonded bromine (area *A*, in Fig. 3b), whereas for physically adsorbed bromine a peak was observed at 67.60 eV (area *A*^’^in Fig. 3b) confirming the bromination of GO^36^. The deconvulated C1s of cellulose exhibited C-C peak at 284.25 eV, C-O peak at 286.01 eV and C-O-C at 288.07 eV (Fig. 3c)^37^. In case of bCel the deconvulated Br 3d reveals peak at 70.01 eV corresponding to covalently bonded bromine while peak at 67.29 eV (Fig. 3d) represents for physically adsorbed bromine which is similar to bGO as discussed above^38^.

Similarly, the deconvoluted N1s peak of qGO (Fig. 4a) clearly showed the existence of two peaks viz., at 402.2 eV and at 399.76 eV, whereas for qCel the peak were at 402 eV for quaternary nitrogen at and 398.92 eV (Fig. 4b) for pyrrolic nitrogen, respectively. Thus the above peaks confirm the quaternization of qGO and qCel, respectively.

**Figure 5:**
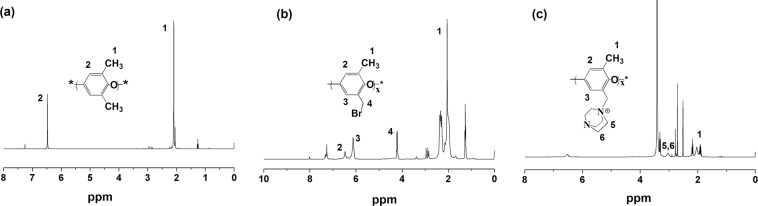
^1^HNMR spectra of (**a**) PPO, (**b**) bPPO, and (**c**) qPPO. (PPO=poly(phenylene oxide), bPPO = brominated poly(phenylene oxide), qPPO = quaternized poly(phenylene oxide)).

The functionalization of PPO was also evaluated by ^1^HNMR, as shown in Fig. 5. The chemical shifts value appeared at 4.3 ppm were assigned to the hydrogens (H-4) of bromo methyl group (-CH_2_Br) group^39^, further indicating successful bromination of PPO. The degree of bromination (DB) was calculated from the peak intensity of bromomethyl and methyl group as shown below;

$$DB=\frac{3\times {I}_{C{H}_{2}}}{2\times {I}_{C{H}_{3}}+3\times {I}_{C{H}_{2}}}$$

Where, *I* is the intensity of the peaks. The degree of bromination of bPPO was 22%. After the quaternization, the peak at 4.3 ppm diminished due to the attachment of DABCO to PPO chain backbone. Furthermore, a characteristic signal corresponding to DABCO at 3.0 ppm also appeared on the qPPO spectrum^40^, additionally implying successful quaternization. The degree of quaternization (DQ) was found to be 76% for qPPO as calculated from the peak intensities of H-5, H-6 and H-1 proton from Fig. 5c ^41^.

### Morphology and structure

**Figure 6:**
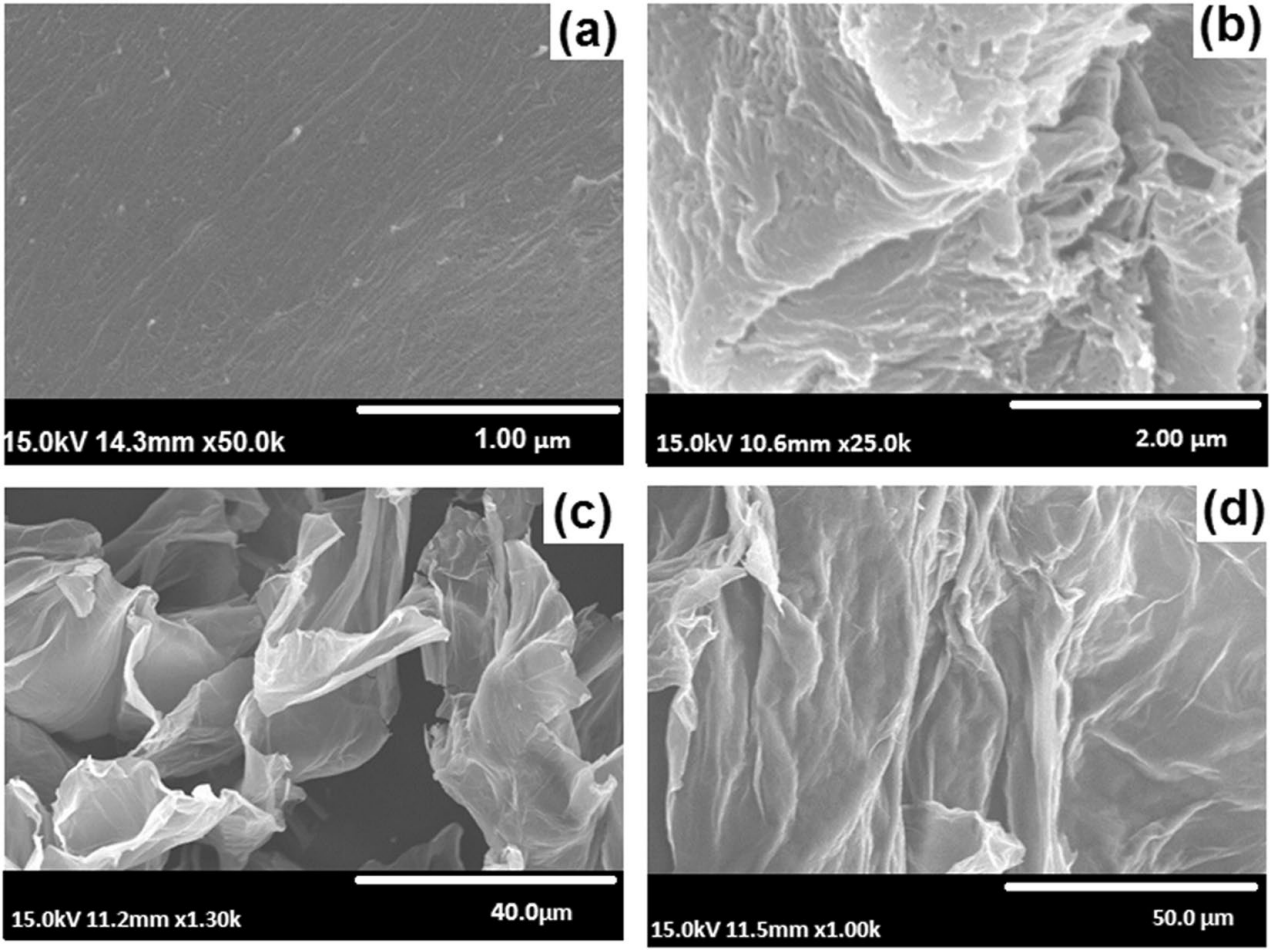
SEM micrographs (**a**) cellulose, (**b**) bCel, (**c**) GO, (**d**) bGO.

**Figure 7:**
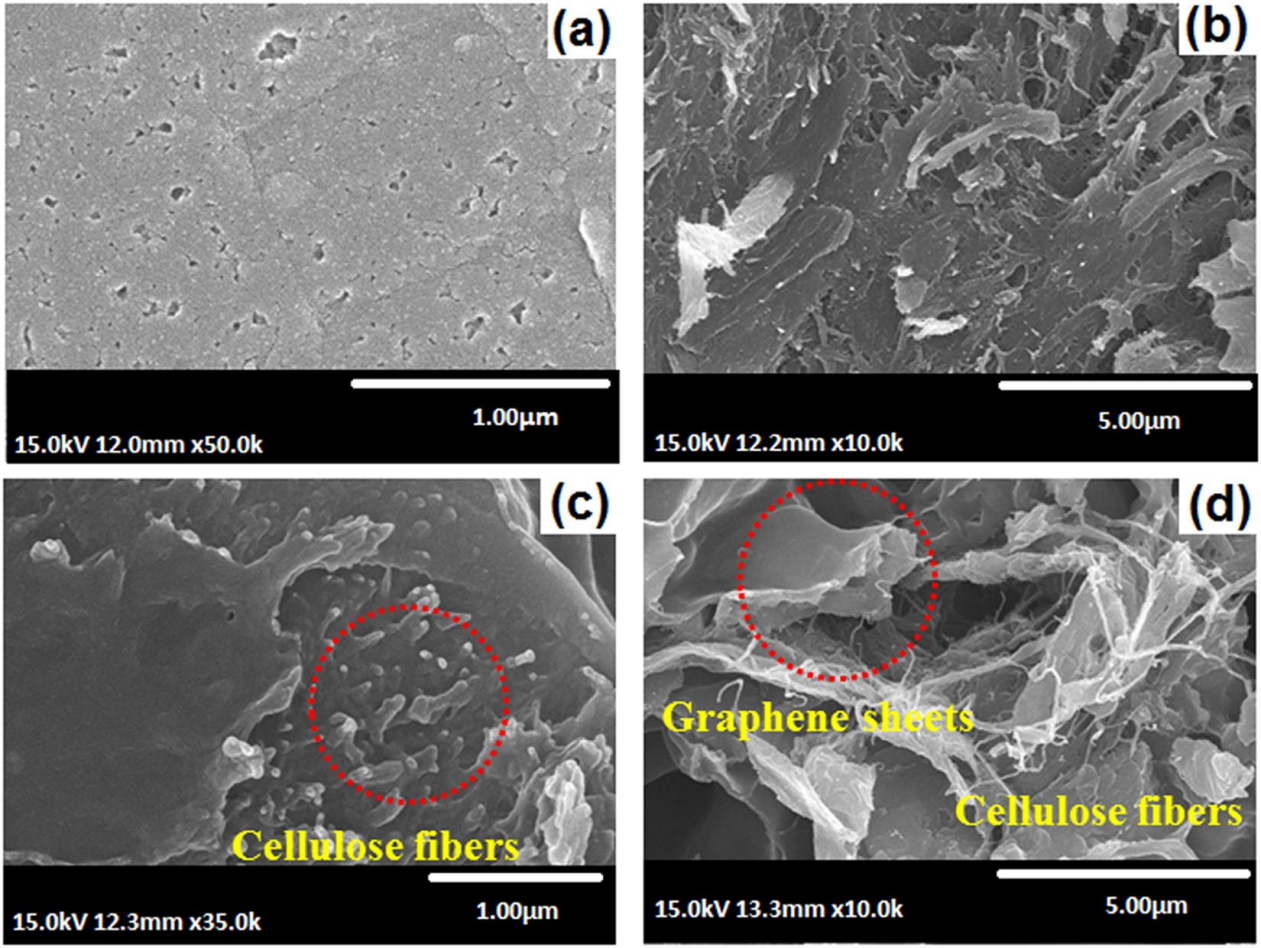
Fractured cross-sectional SEM micrographs of (**a**) PPO/C-0/G-0, and (**b**–**d**) PPO/C-1/G-1with different magnification.

Figure 6 shows SEM images of cellulose, bCel, GO, and bGO. Pristine cellulose was seemingly fibrous, after bromination, the structural features remained intact in bCel (Fig. 6a,b). Similarly, GO, which exhibited a sheet like morphology, showed no variation after subsequent reactions (Fig. 6c,d). Thus, the structure of both the fillers remained intact which was favorable for good interaction with the polymer matrix. Further, the EDX spectra (Supplementary Fig. S1) and the elemental composition analysis (Supplementary Table S1) revealed that the elements were close to those of precursors, implying a successful functionalization of cellulose and GO. Figure 7a–d represents fractured cross-sectional images of the membrane samples. The surface feature of the pristine qPPO (PPO/C-0/G-0) was seemingly fragile with cracks and pots (Fig. 7a). On the other hand, qPPO with filler (PPO/C-1/G-1) exhibited a dense and rough surface with pores (Fig. 7b). The high magnified SEM images of PPO/C-1/G-1 showed that cellulose fibers were well embedded within the polymer matrix (Fig. 6c), and GO sheets and cellulose fiber (100 nm of average diameter) were uniformly dispersed in the matrix polymer (Fig. 7d), probably due to a good dispersibility of qCel and qGO in NMP solution as revealed in a dispersion stability test (Supplementary Fig. S2). The result, therefore, suggested that the good dispersion of cellulose and GO could be obtained by quarternization. It is noteworthy to mention that good dispersibility of quarternized cellulose and GO might provide high interfacial interaction which aided in an exfoliated type structures. Consequently, during membrane casting and evaporation of the solvent, the fillers had lesser tendency to agglomerate; this altogether facilitates a uniform distribution of the fillers in the membrane matrix.

**Figure 8:**
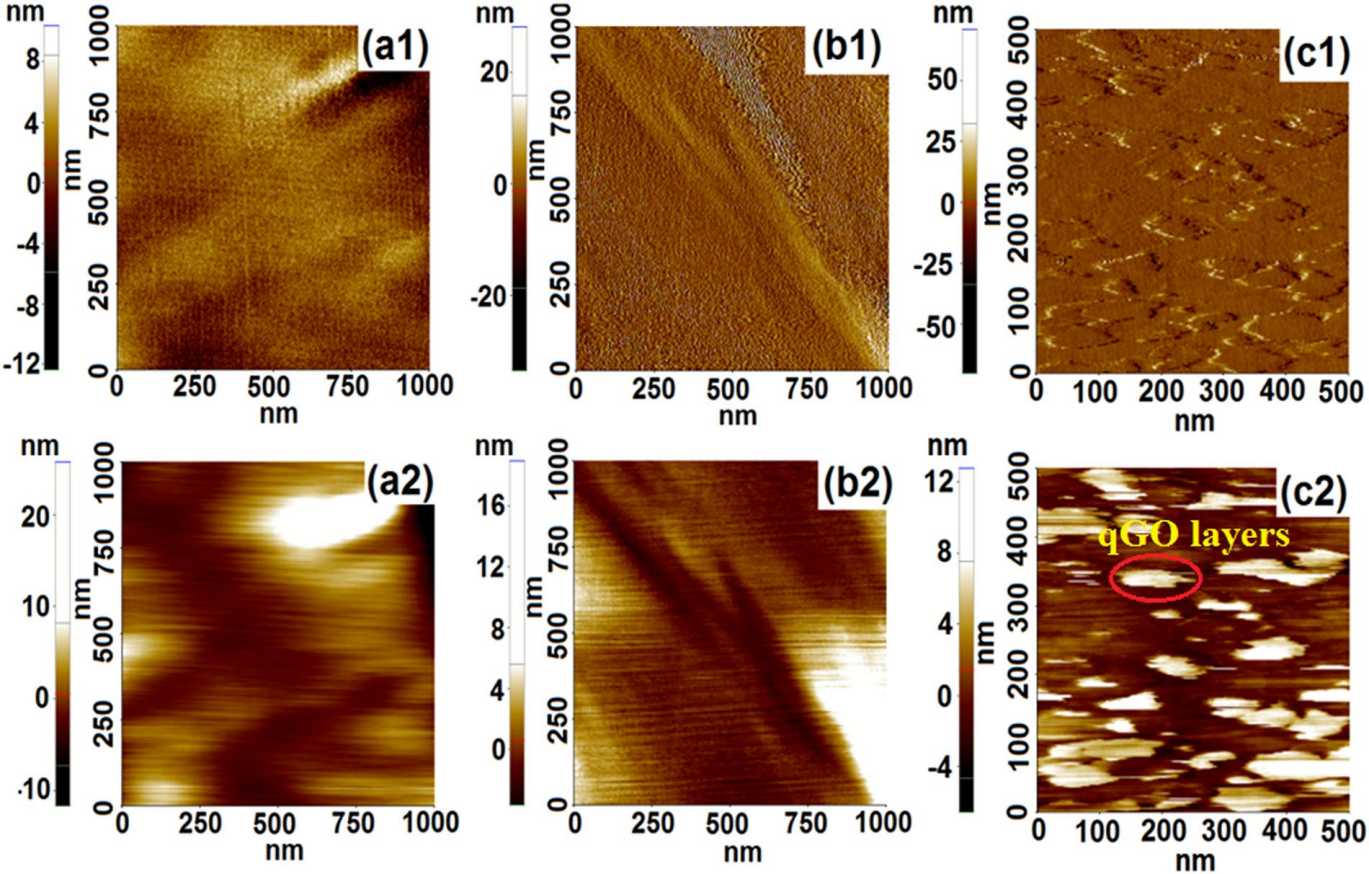
AFM height (**a1**) PPO/C-0/G-0, (**b1**) PPO/C-1/G-0, and (**c1**) PPO/C-1/G-1 and phase images of (**a2**) PPO/C-0/G-0, (**b2**) PPO/C-1/G-0, and (**c2**) PPO/C-1/G-1.

The height and phase AFM image of PPO/C-0/G-0, PPO/C-1/G-0, and PPO/C-1/G-1 are presented in Fig. 8. A phase-aggregated morphology at microscopic scale was confirmed in the cross-linked PPO-based membranes from the separation of hydrophobic and hydrophilic domains in the phase AFM images; the darker and light region can be ascribed to hydrophilic and hydrophobic domain, respectively^42^. The phase/height AFM images of PPO/C-1/G-1 (Fig. 8c-1 and Fig. 8c-2) displayed the typical GO nanosheet morphology with high contrast. The qPPO chains were absorbed onto the qGO sheets, indicating a good compatibility and adhesion with the PPO matrix probably owing to crosslinking which prevented the agglomeration and stacking of the GO sheets. Due to the uniform distribution of the qGO nanosheets, it is expected that the ionic clusters will form a continuous hydrophilic channels as depicted in Fig. S3, resulting in low barrier to the hydroxide mobility as observed later in ion conductivity measurements.

### Ion exchange capacity, Water uptake, Swelling ratio, and Ionic conductivity

Ion exchange capacity (*IEC*) reflects the exchangeable groups in the membranes which plays a crucial role in water absorption and ionic conductivity. The *IEC* values of the different membranes are presented in Table 1. The pristine qPPO (PPO/C-0/G-0) displayed an *IEC* of 0.85 mmol/g which was close to that of the commercial Nafion 117 (0.9 mmol/g)^43^. The *IEC* value increased to 1.12 mmol/g by the addition of cellulose (PPO/C-1/G-0), and further increased to 2.64 mmol/g by the addition of cellulose and GO as hybrid filler (PPO/C-1/G-1). The *IEC* value varied with cellulose and GO contents; however, it was more dependent on GO content. In case of qGO, higher content of nitrogen reflected more quaternary groups as compared to cellulose (Table S1). Thus, this resulted in more cationic content in the membranes on addition of qGO translating into higher IEC. On the other hand, the *IEC* was observed to decrease with increase in the cellulose content. This might be due to the increased crosslinking density, which reduced the access of the charged ions located at the core^44^. The water uptake (*WU*) is another important parameter that influences the ionic conductivity and mechanical properties of the membranes. The *WU* values also considerably increased from 25.3 to 40.5% by the addition of cellulose (PPO/C-1/G-0), and further to 89.0% by the hybrid cellulose and GO filler (PPO/C-1/G-1), as observed similarly for *IEC*. This indicated that IEC played an affirmative role in *WU* because a suitable water uptake allowed ion clustering, thereby, allowing better ion transport pathways. A high *WU* can induce greater swelling of membranes, affecting membrane’s dimensional stability. However, no significant increase of *SR* values was observed by the presence of cellulose and GO; the *SR* (*SR*_*ip*_/*SR*_*tp*_) value of PPO/C-0/G-0 and PPO/C-1/G-1 were 12.1/14.9 and 17.2/14.9%, respectively.

**Table 1:** Properties of as synthesized composite membranes measured at room temperature.

Membrane sample	*IEC* (meqv/g)	*WU* (%)	*SR*_*ip*_ (%)	*SR*_*tp*_ (%)	*Gel fraction* (%)
qPPO/C-0/G-0	0.85	25.34	12.07	14.94	75
qPPO/C-1/G-0	1.12	40.52	11.56	18.18	95
qPPO/C-0.5/G-1	2.35	78.87	11.49	15.24	88
qPPO/C-1/G-1	2.64	88.97	17.21	14.28	92
qPPO/C-2/G-0.5	2.09	86.19	22.88	21.83	87
qPPO/C-3/G-0.5	1.82	88.13	25.55	30.80	85

The *WU* and swelling characteristics of the composites membranes were further studied at different temperature as shown in Supplementary Fig. S4 and S5. The effect of qCel and qGO on the *WU* and *SR* at higher temperatures (60 and 80 °C) remained the same as those observed at room temperature. However, both the *WU* and *SR* values increased with temperature. However, none of the composite membranes exhibited any rupture. The effect of crosslinking on the dimensional stability was further examined by gel fraction measurements. As shown in Table 2, gel fraction increased from 75% to 92% by the addition of cellulose and GO. Furthermore, qPPO membrane without crosslinking with DBB dissolved completely in NMP solution in 4 h at room temperature. In contrast, the crosslinked membrane remained intact with fewer tendencies for deformation even after heating at 70 °C for 72 h as shown in Supplementary Fig. S6, indicating the dimensional stability was considerably enhanced by crosslinking reaction.

**Table 2:** Hydration number (λ) and Ionic conductivities of as-synthesized composite membranes at room temperature.

Membrane sample	λ (OH^−^)	σ_Br-_	σ_xy_	σ_z_	σ_xy_/σ_z_
qPPO/C-0/G-0	16.56	9.50	20.20	19.50	1.03
qPPO/C-1/G-0	20.07	15.88	31.93	29.41	1.08
qPPO/C-0.5/G-1	18.64	25.68	79.71	73.50	1.08
qPPO/C-1/G-1	18.74	36.89	114.64	112.89	1.01
qPPO/C-2/G-0.5	22.91	17.82	60.23	46.74	1.28
qPPO/C-3/G-0.5	24.02	16.88	68.92	46.10	1.49

**Figure 9:**
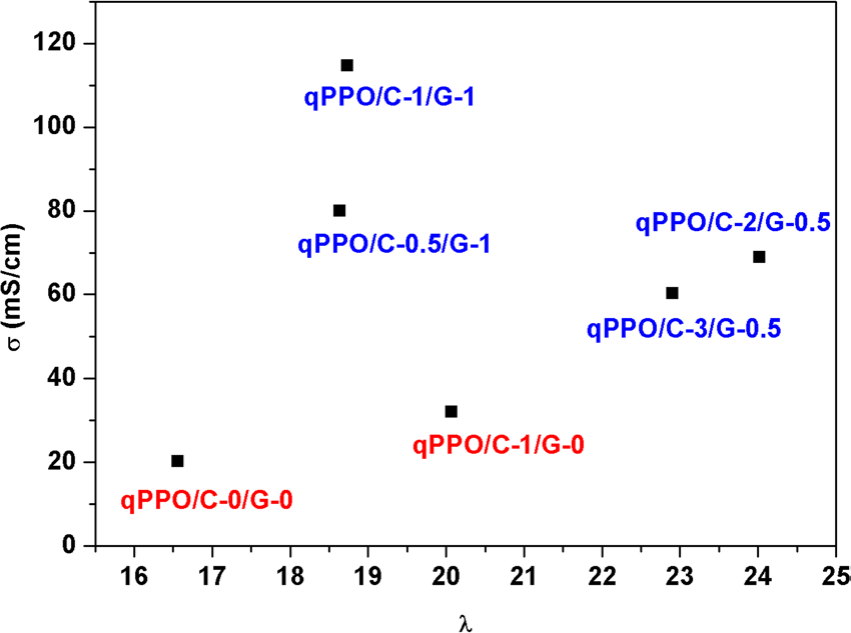
Plot of hydroxyl conductivity vs. hydration number (λ) of the different composite membranes.

The ionic conductivities of the composite membranes were measured by the in-plane method as summarized in Table 2. The bromide conductivity of pristine qPPO (PPO/C-0/G-0) was 9.50 mS/cm. The bromide conductivity increased ~1.6 folds for cellulose composited PPO (PPO/C-1/G-0), and further enhanced by cellulose and GO hybrid composition. After exchange reaction, the hydroxide conductivity of the pristine qPPO exhibited an in-plane conductivity of 20.20 mS/cm and a through-plane conductivity of 19.50 mS/cm. The ionic conductivity was plotted as a function of hydration number (λ) in order to have a better comparison as shown in Fig. 9. The introduction of qGO and qCel into qPPO increased the hydration number as depicted in Table 2, due to the increase in the number of water molecules per ionic sites. In case of qPPO/C-1/G-1 and qPPO/C-0.5/G-1, λ were 18.64 and 18.74 with ionic conductivity of 114.64 and 79.71 mS/cm, respectively. Although, the λ value for qPPO/C-1/G-1 and qPPO/C-0.5/G-1 were lower than those for qPPO/C-2/G-0.5 and qPPO/C-3/G-0.5, the high hydroxyl conductivity obtained for qPPO/C-1/G-1 and qPPO/C-0.5/G-1 might be resulted from better ionic clustering forming interconnected ionic channels. On the other hand, the high λ resulted in dilution of the charge carriers, which might affect their ionic transport. The degree of anisotropy (σ_xy_/σ_z_) of the composite membrane were in the range of 1.08-1.49, indicating that the fillers were uniformly distributed in lateral and perpendicular direction with respect to the core. Similar result was obtained in our previous work^44^. The probable reasons for such observation might be due to the homogeneous dispersion of the fillers in the polymer matrix resulting in uniform charge distribution^45^. Although further systematic study should be needed, the optimum qCel and qGO loading was found to be 1 wt% (for both) from the limited experiments as shown in Table 2.

The temperature dependency of the composite membrane on the hydroxyl conductivity was examined as shown in Supplementary Fig. S7. For all membranes, the conductivities increased with temperature as expected. At higher temperature, the qGO/qC composited PPO membranes had greater *WU* values, which enhanced polymer chain flexibility and led to rapid ion migration and higher ionic diffusivity. In addition, the composite membranes exhibited stable hydroxyl conductivity at 80 °C.

A performance comparison of the membranes reported previously, as shown in Table 3, revealed that the qPPO/qC/qGO composites developed in this study exhibited considerably higher anion conductivity at room temperature (25 °C) even more at an elevated temperature (80 °C).

**Table 3:** Membrane performance data from literature.

Membranes types	IEC (mmol/g)	WU(%)	*σ*_OH_^−^ at RT^ǂ^ (mS cm^−1^)	*σ*_OH_^−^ > RT (mS cm^−1^)	Reference
QPSU/GO/IM-3	3.44	105.55	~42.0@25 °C	102.10@100 °C	^46^
QPAES/7.5% ZrO_2_	1.74	23.8	19.8@20 °C	41.40@80 °C	^47^
ImPSF/QAB-OMS-1	~1.74	24.27	38.33@30 °C	64.50@80 °C	^48^
ImPPO/IL-GO-1.0%	2.12	50.50	~40.0@30 °C	78.50@80 °C	^25^
40-Im-SiO_2_/TA-PPO	3.15	145	~36.0@30 °C	105.0@80 °C	^49^
qPPO/C-1/G-1	2.64	88.97	114.64@25 °C	215.66 @80 °C	This work
qPPO/C-0.5/G-1	2.35	78.87	79@25 °C	157.32@80 °C	This work

^ǂ^RT = room temperature; *QPSF = quaternized polysulfone; ZrO_2_ = zirconium oxide; ImPSF = imidazolium functionalized polysulfone; QAB-OMS = quaternary ammonium brush- organic microspheres; ImPPO = imidazolium functionalized polyphenylene oxide; IL-GO = ionic liquid functionalized graphene oxide; Im-SiO_2_ = imidazolium functionalized-SiO_2_; TA-PPO = triple-ammonium side chain-polyphenylene oxide.

### Alkaline stability

**Figure 10:**
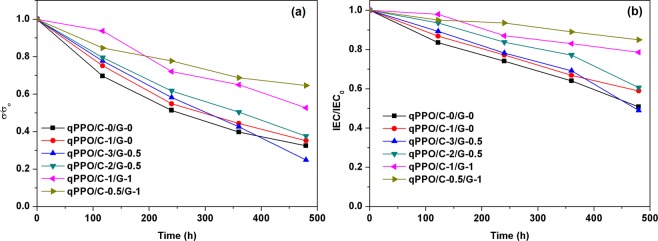
(**a**) Change of relative hydroxyl conductivity and (**b**) *IEC* of the composite membranes after immersion in 3 M KOH at room temperatures.

The stability of the composite membranes was evaluated for long time operation in fuel cells. The membranes were immersed in 3 M KOH for 480 h, and the stability was checked after 120 h by determining the hydroxyl conductivity and *IEC* (Fig. 10). It was seen that the stability of the membrane decreased with the increase in the qCel content, whereas the membranes demonstrated greater stability at higher content of qGO. This might be due to the fact that cellulose contained oxygenated functionalities which might be susceptible for nucleophilic attack rendering its degradation and, in contrast, the graphitic framework of GO was very stable against the alkaline environment. Pristine qPPO retained about 32% of its conductivity after 480 h, whereas qPPO/C-0.5/G-1 retained 64% of its original conductivity. The *IEC* of the composite membranes followed a similar trend. qPPO/C-0.5/G-1 exhibited the highest stability. The addition of qCel and qGO hybrid filler might reduce the free volume in the membrane, and the crosslinking the compositions restricted the free motion of the polymer chains, preventing degradation of the composite membrane by the attack of the OH^−^ anion^25,28^.

## Conclusion

In this study, we have demonstrated a new strategy to synthesize an anion exchange composite membrane. The as-prepared qCel and qGO hybrid nanofiller-composited qPPO membrane exhibited considerably enhanced ionic conductivity, alkaline stability, and mechanical property as compared to the pristine qPPO membrane. The hybrid fillers were uniformly distributed in lateral and perpendicular direction with respect to the core, mainly due to the quaternization of each component. The qCel and qGO hybrid nanofiller might provide more interconnected ionic channels, enhancing the ion conductivity of the composite membrane. The highest hydroxyl conductivity of 114.64 mS/cm at 25 °C and 215 mS cm^−1^ at 80 °C was achieved for qPPO/C-1/G-1, which is much higher than those of previous reports. Furthermore, the mechanical and chemical stability were also significantly enhanced by the addition of qCel and qGO, mainly because the nanofiller might reduce the reduction of free volume in the membrane, and the crosslinking restricted free motion of the polymer chains. The results suggested that the introduction of cellulose and GO hybrid filler into PPO matrix is a promising approach for designing high performance anion-exchange membranes.

## Methods

### Materials

Cellulose, Graphite flakes, 1,4-Diazabicyclo[2.2.2]octane (DABCO), poly(phenylene oxide) (PPO), lithium bromide (LiBr), N-bromosuccinimide (NBS), N-methyl-2-pyrrolidone (NMP), 1,4-dibromobutane (DBB), N,N-dimethylacetamide (DMA), N.N-dimethylformide (DMF), and 2,2′-azobisisobutyronitrile (ABN) were purchased from Sigma Aldrich (Korea). Graphene oxide was synthesized as described in a previous report^33^.

### Bromination and quaternization of graphene oxide

GO was brominated as described in a previous report^34^. Briefly, a dispersion of GO (13 mg/mL) in 75 mL of NMP was prepared by sonication and mechanical stirring, and then LiBr (22 g) was added and stirred for 30 min. Afterward, NBS (2 g) and Ph_3_P (3 g) were added and stirred vigorously for 4 h at 80 °C under inert atmosphere. The reaction content was poured into 100 mL of de-ionized (DI) water, and a blackish precipitate was collected after filtration. The filtrate was washed repeatedly with DI water and acetone to remove any traces of unreacted chemicals. The product was finally dialyzed against a phosphate buffer solution (PBS) and then lyophilized to obtain brominated graphene oxide (bGO).

The bGO (0.1 g) was quaternized by reacting with DABCO (0.6 g) in 20 mL NMP for 72 h at 70 °C. The product was suspended in toluene and filtered. The quaternized graphene oxide (qGO) was then obtained by washing with diethyl ether and ethyl acetate and drying in a vacuum oven for overnight at 50 °C.

### Bromination and quaternization of cellulose

Cellulose (1 g) was first dispersed in 35 mL of DMA and 1.25 g of LiBr was added and stirred for 30 min. Afterward, NBS (2.1 g) and Ph_3_P (3.2 g) were added and stirred for 1 h at 80 °C. The brominated product was dispersed in DI water and filtered. The filtrate was repeatedly washed with DI water and ethanol. After then, it was lyophilized to obtain brominated cellulose (bCel).

The bCel (0.5 g) was dissolved in DMF and then 1.2 g of DABCO was added under vigorous stirring. The reaction was carried out for 24 h, after which the whole content was poured in diethyl ether and washed with diethyl ether and ethyl acetate repeatedly. Finally, the powdered material obtained after purification was lyophilized to obtained quaternized cellulose (qCel). The schematic of the cellulose quarternization reaction is presented in Supplementary Scheme 1.

### Bromination and quaternization of PPO

The typical bromination of PPO is shown in Supplementary Scheme S2; wherein, PPO (100 mmol) was dissolve in chlorobenzene (60 mL), and then, to this solution, NBS (8.9 g) and ABN (0.5 g) were added. The reaction was allowed to continue for 12 h under continuous N_2_ purging. After cooling, the reaction mixture was precipitated in a 1 L of ethanol. The precipitate was washed with ethanol several times. Subsequently, the precipitate was re-dissolved in chloroform and precipitated in ethanol. The obtained precipitate was then dried in a vacuum oven at 45 °C for overnight to obtain brominated PPO (bPPO).

The bPPO (1 g) was dissolved in 20 mL NMP. To this dispersion, 1.4 g DABCO was then added and stirred at 70 °C for 24 h. The reaction content was precipitated in diethyl ethyl (100 mL) and washed with diethyl ether and ethyl acetate. The obtained product was then dried in a vacuum oven for overnight at 45 °C to obtain quaternized PPO (qPPO).

### Composite and membrane formation

The schematic representation of the formation of the nanocomposite is shown in Supplementary Scheme 2. Firstly, a 17 wt% solution of qPPO in NMP was prepared. It was then mixed with qGO and qCel at different weight ratios [i.e., (qGO + qCel)/qPPO (wt/wt) = 0, 0.15, 0.25, 1 and 2] under vigorous stirring for 12 h at 70 °C. The qGO and qCel were dispersed separately in NMP before adding to qPPO solution. The qPPO solution containing qGO and qCel was heated with the addition of DBB as crosslinking agent at 70 °C for 20 min to obtain a pre-gelled liquid. The pre-gelled solution was poured into a glass petridish and dried in an air circulating oven at 80 °C. After then, the solvent was evaporated for 6 h. The resulting composite membrane was transferred into a vacuum oven and dried at 80 °C until a constant weight of the membrane was obtained. The membranes with different composition were synthesized and denoted as summarized in Table 4. The membranes were coded as PPO/C-*x*/G-*y* (C = qCel; G = qGO), where *x* and y are wt% of qCel and qGO per qPPO in the hybrid composite membrane.

**Table 4:** Composition in various membrane samples.

Membrane samples	qPPO, g	qCel, g	qGO, g	qGO/qCel g/g	(qGO + qCel) /qPPO, g/g
qPPO/C-0/G-0	100	0	0	—	0
qPPO/C-1/G-0	100	1.0	0	0	1
qPPO/C-0.5/G-1	100	0.5	1.0	2.0	1.5
qPPO/C-1/G-1	100	1.0	1.0	1.0	2.0
qPPO/C-2/G-0.5	100	2.0	0.5	0.25	2.5
qPPO/C-3/G-0.5	100	3.0	0.5	0.17	3.5

### Analysis

A Fourior transform infra spectrometer (FTIR, JASCO FT-IR 300E) was used to record the FTIR spectra of the samples. The structure and the degree of bromination of PPO, bPPO and qPPO were studied by 400 MHz FT-NMR Spectrometer (Avance II, Bruker Biospin). The X-ray photoelectron spectra of GO, BrGO and QGO were obtained by X-ray photoelectron spectrophotometer (XPS, K-alpha, Thermo VG, U.K.) employing monochromated Al X-ray source (Al Kα line: 1486.6 eV). The morphological characterization of the samples was carried out by scanning electron microscope (SEM, Hitachi S-4700, Japan). The architecture-morphology-properties of the membranes were characterized by atomic force microscopy (AFM, XE-150, Park system, Korea).

Ion conductivity (σ) (σ_*xy*_ = in-plane, σ_*z*_ = through-plane), ion exchange capacity (IEC), water uptake (*WU*), swelling ratio (*SR*) (*SR*_*wy*_ = in-plane, *SR*_*z*_ = through-plane), and gel fraction (GF) were measured according to the procedure as described in a previous report^45^; detailed procedure is summarized in Supplementary Information.

## Supplementary information

SOM

## Data Availability

The datasets generated during and/or analysed during the current study are available from the corresponding author on reasonable request.
