# Supplementary material for: Quaternized cellulose and graphene oxide crosslinked polyphenylene oxide based anion exchange membrane
Source: Sci Rep. 2019 Jul 2;9:9572. doi: 10.1038/s41598-019-45947-w (PMC6606628; doi:10.1038/s41598-019-45947-w)
Supplement: Supplementary file 1 — SOM [file 41598_2019_45947_MOESM1_ESM.docx]

***Electronic Supplementary Information for:***

**Quaternized cellulose and graphene oxide crosslinked polyphenylene oxide based anion exchange membrane**

Gautam Das^1^, Bang Ju Park^2^, Jihyeon Kim^1^, Dongho Kang^1^, and Hyon Hee Yoon^1^*

^1^Department of Chemical and Biological Engineering, Gachon University, Seongnam, Gyeonggi-do 13120, Republic of Korea

^2^Department of Electronic Engineering, Gachon University, Seongnam, Gyeonggi-do 13120, Republic of Korea

**Ion exchange capacity (IEC), water uptake (WU) and swelling ratio (SR).** The IEC measurement was adopted from previously published reports [1]. Briefly, the hydroxide exchange membranes were equilibrated with standard HCl solutions (0.01 mol/L ). Then the OH− ions liberated was determined by standard NaOH (0.01 mol/L) solution using acid-base titration. The ion-exchange capacity (IEC; mmol/g) of the membrane was calculated by Eq. 1, where V_a_ is the volume of the blank samples, V_b_ is the volume consumed for the test sample, C_NaOH_ is the concentration of NaOH and M­_dry_ is the mass of the dried samples.:

IEC=((V_a_-V_b_)×C_NaOH_)/M_dry_ (4)

The WU and SR were carried out as follows. The exchanged membrane samples were soaked in deionized water for 24 h at different temperature, the membranes were taken out and the surface water was wiped, and the weight and the dimensions were measured. The membranes were heated at 50 °C placed in a vacuum oven until a constant weight was obtained. The WU and the SR were determined by following the equation as given below:

$WU=\left( \frac{W_{2}-W_{1}}{W_{1}} \right)\times100$ (2)

${SR}_{ip} =\frac{L_{wet}-L_{dry}}{L_{dry}}$) (3)

$\mathrm{SR}_{tp}= \frac{T_{wet}-T_{dry}}{T_{dry}}$ (4)

In the above equations *W_1_* and *W_2_* are the weight of the wet and dry samples. In Eq. 3, *SR_ip_* is the in-plane with *L_wet_,* and *L_dry_* representing the width in wet and dry conditions. Similarly, *SR_tp_* in Eq. 4 represents the through-plane swelling ratio and *T_wet_* and *T_dry_* are the thickness or the membranes in wet and in dry conditions, respectively.

**Ionic conductivity.** A two electrode home-made probe was used to measure the through-plane (σ*_z_*) and in-plane (σ*_xy_*) ion conductivity by Solartron 1255B and 1287 frequency response analyzer. The Nyquist plots were obtained in a frequency range of 100 kHz to 7 MHz with amplitude of 50 mV under N_2_ atmosphere with a 100% humidity. The anion conductivity was calculated by the following equation:

σ_xy_=*d*/(R×A) (5)

σ_z_=*l*/(R×A)

Where d (cm) is the distance between the electrodes and *l* is the thickness of the membranes; R (ohm) is the resistance of the membrane and A (cm^2^) is the area of the membrane.

**Gel fraction.** The gel fraction was determined heating the membranes cut into specific size in highly polar solvents such as N-methyl pyrrolidone (NMP) at 70 °C for 72 h. The weight before and after the immersion were determined to calculate the gel fraction.

Table S1. Elemental composition of nanofillers as measured by EDX analysis.

| **Samples** | **Elemental composition (%)** | | | |
| --- | --- | --- | --- | --- |
|  | **N** | **C** | **H** | **S** |
| Cellulose | 0.00 | 39.63 | 6.828 | 0.100 |
| qCel | 1.79 | 42.11 | 6.822 | 0.759 |
| GO | 0.05 | 38.87 | 3.299 | 1.901 |
| QGO | 9.13 | 53.80 | 6.441 | 0.356 |


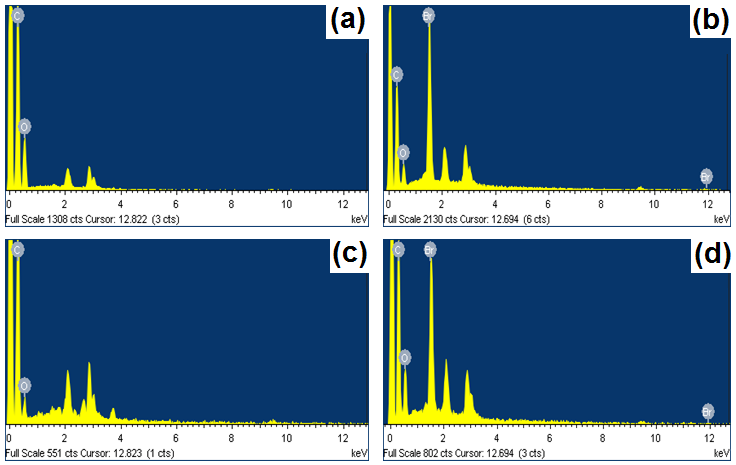


Figure S1. EDX spectra of (a) cellulose, (b) bCel, (c) GO, and (d) bGO.

*
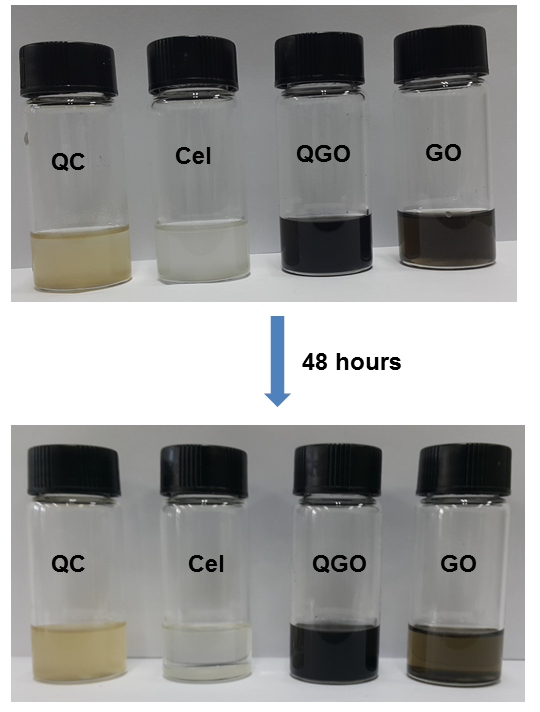
*

Figure S2. Dispersion stability of functionalized Cel and GO.


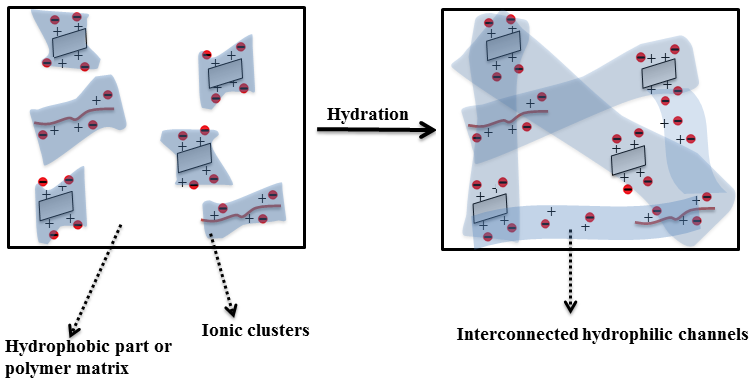


Figure S3. Schematics of possible ionic channels in the AEMs.


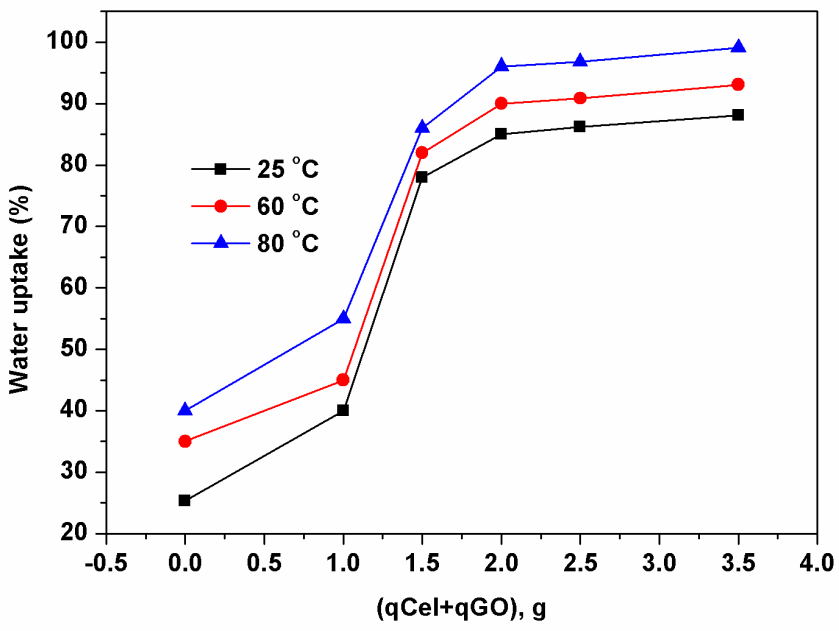


Figure S4. Water uptake of qPPO composite membranes with temperature at different qCel+qGO content.


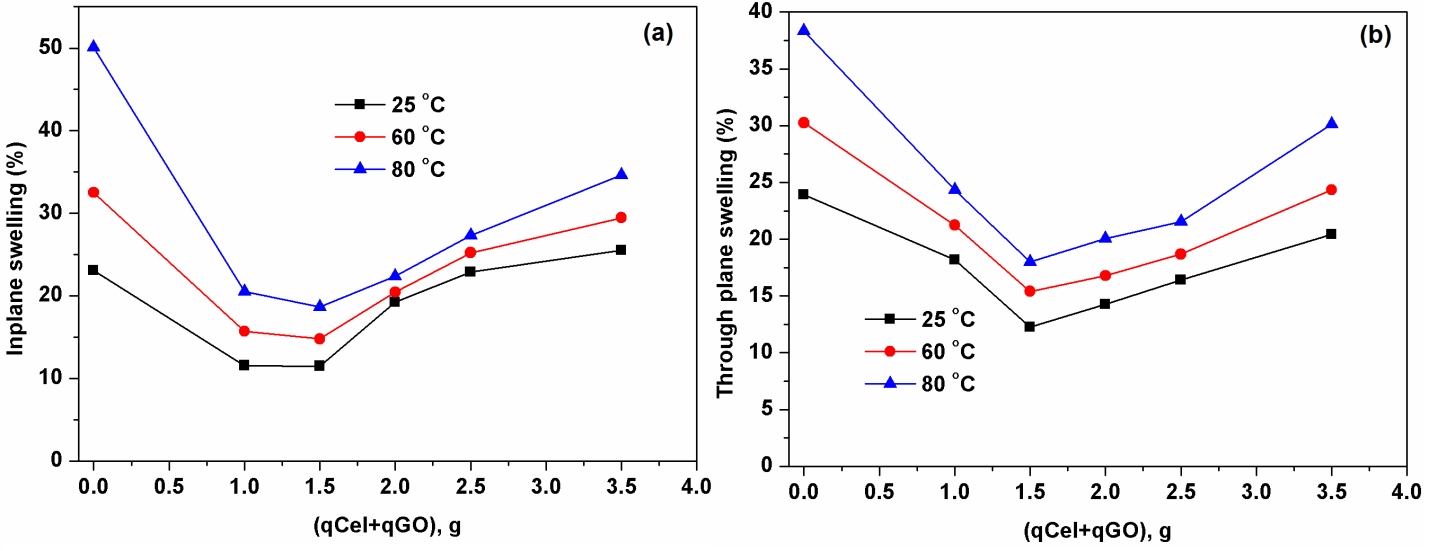
Figure S5. Swelling characteristics of qPPO composite membranes with temperature at different qCel+qGO content.

**
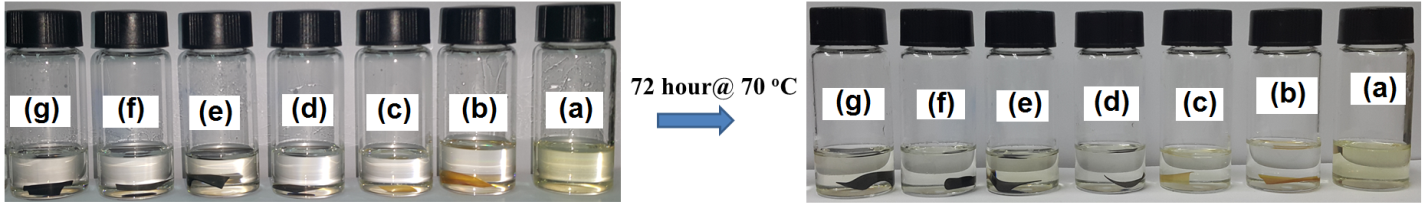
**

Figure S6. Optical images of the membranes (a) qPPO, (b) qPPO/DBB, (c) qPPO/C-1/G-0, (d) qPPO/C-3/G-0.5, (e) qPPO/C-0.5/G-1, (f) qPPO/C-2/G-0.5, (g) qPPO/C-1/G-1 before and after immersion in NMP.


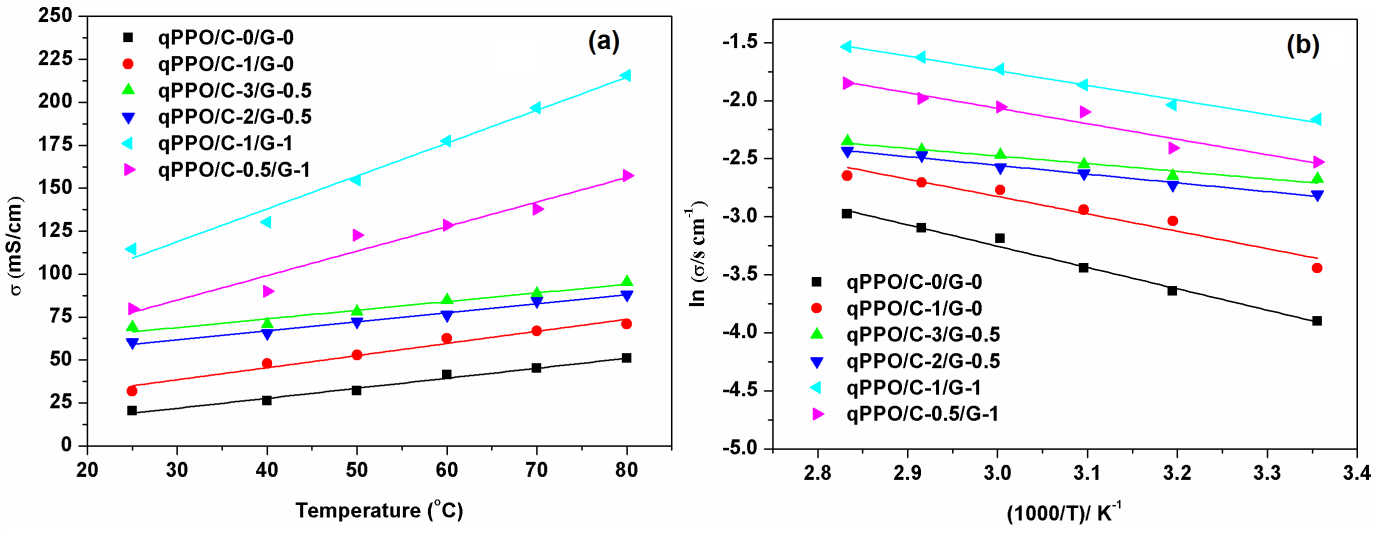


Figure S7. Hydroxyl conductivity and Arrhenius plot for different composite membranes.

*

*

Scheme S1. Quaternization of cellulose.


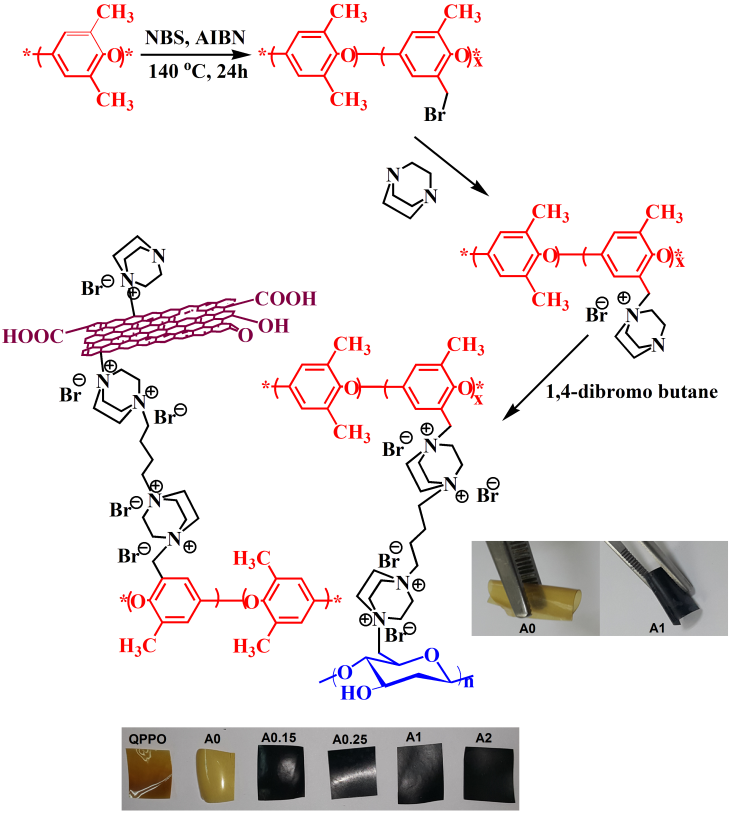


Scheme 2. Schematics for the synthesis of composite membranes.
